# Bilateral lower extremity ulcerations, less is more

**DOI:** 10.1002/ccr3.1926

**Published:** 2019-01-09

**Authors:** Kathryn Mooneyham Potter, Garrett Ruth, Nila S. Radhakrishnan

**Affiliations:** ^1^ Division of Dermatology University of Florida Gainesville Florida; ^2^ Department of Medicine University of Florida Gainesville Florida; ^3^ Division of Hospital Medicine University of Florida Gainesville Florida

**Keywords:** biologics, lowered extremity ulcers, pathergy, pyoderma gangrenosum

## Abstract

The differential diagnosis of lower extremity ulcers must be broad since debridement can be harmful in certain conditions such as pyoderma gangrenosum. Biologic agents may be helpful in the treatment of pyoderma gangrenosum.

## 1 PRESENTATION

A 24‐year‐old female with a past medical history of SLE, rheumatoid arthritis, deep vein thromboses, pulmonary emboli, and interstitial lung disease presented to the emergency room with complaints of worsening bilateral lower leg ulcers for 6 months. The lesions started out as small papules that began to ulcerate. Outpatient, she was initially treated with topical steroids early in her course and noted mild improvement. However, she then saw another physician who started her on oral antibiotics to treat for superficial infection. Despite multiple rounds of treatment and aggressive wound care including debridement, the lesions continued to enlarge. She has been on chronic steroids and is currently on 10 mg prednisone daily. Her condition has worsened to the point she is no longer able to ambulate due to pain, which is why she presented to the Emergency Room.

## 2 ASSESSMENT

On admission, vital signs were normal. Patient is cushingoid. The remainder of the physical examination is unremarkable. Laboratories show WBC of 13.9 with normal differential; ESR 88 and CRP 93.4. X‐ray of left lower extremity was significant for periosteal elevation. MRI showed soft tissue inflammation, no evidence of osteomyelitis.

**Figure 1:**
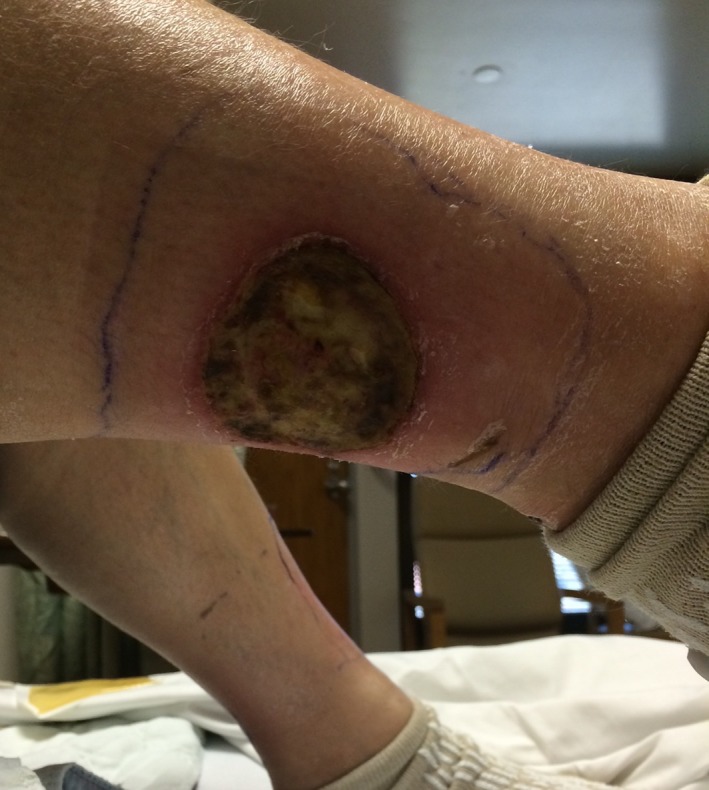
Ulceration

**Figure 2:**
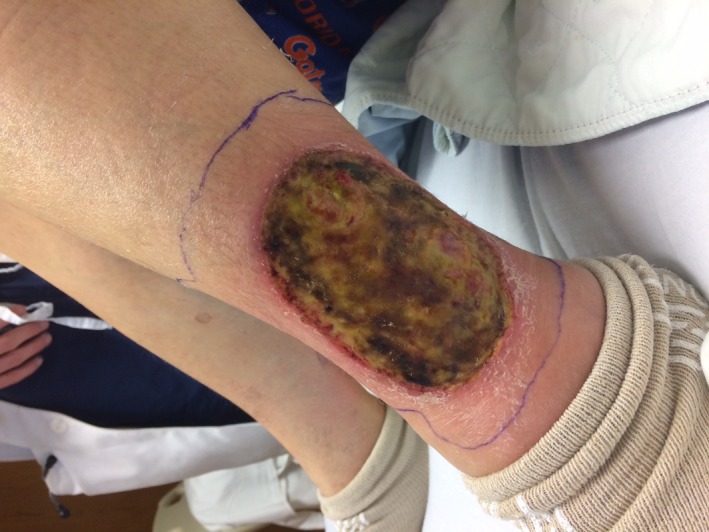
Ulceration

The ulcerations on bilateral lower extremities are shown in Figures 1 and 2.

## 3 DIAGNOSIS

The differential diagnosis in a patient with bilateral ulcerative lesions includes infection such as cellulitis and osteomyelitis. Vasculitis and inflammatory lesions resulting from a rheumatoid arthritis flare are also included in the differential. This patient presented after multiple doses of antibiotics and debridement which actually lead to worsening ulceration. This is typical of pyoderma gangrenosum, showing pathergy, or minor trauma leading to ulcers that are resistant to healing.^1^ On admission to the Emergency Room, an X‐ray of her left leg showed mild periosteal elevation concerning for osteomyelitis, so an MRI was performed. This would not have been necessary had the X‐ray not been concerning. The two major criteria for the diagnosis of pyoderma gangrenosum include rapid progression of a painful ulcer and exclusion of other causes of cutaneous ulceration.^1^

Pyoderma gangrenosum is an uncommon neutrophilic dermatosis presenting as an inflammatory ulcerative lesion of the skin. Typically, the lesions begin with pustules, plaques, red papules, or nodules that characteristically ulcerate with rapid expansion. The ulcers commonly have violaceous, undermined borders.^1^ In a retrospective review of 100 patients, over 50% of those diagnosed with pyoderma gangrenosum had associated systemic disease including inflammatory bowel disease (34%), arthropathies (19%), and hematologic malignancies (21%).^2^ Pyoderma gangrenosum is a diagnosis of exclusion which is made clinically. Suggestive factors include rapid progression of a painful ulcer, history suggestive of pathergy, association with systemic inflammatory disorders, and rapid response to steroids. Biopsy not used to make the diagnosis of pyoderma gangrenosum.^1^

In our patient, biopsy was performed to exclude other causes for ulceration. Biopsy showed “sparse, nonspecific dermal, and subcutaneous inflammatory infiltrate with angioplasia and thickened pannicular septa.”

## 4 MANAGEMENT

Our patient was started on 60 mg of oral prednisone daily. Soon after, she was started on Adalimumab (Humira) 40 mg subcutaneously every other week.

Although the pathophysiology of pyoderma gangrenosum is unknown, it is associated with inflammatory diseases that many times respond to TNF‐alpha blockade. A case series that retrospectively collected data on 13 patients with inflammatory bowel disease and pyoderma gangrenosum treated with Infliximab showed that all thirteen patients responded to therapy.^3^ Adalimumab is more convenient for patients than Infliximab because of the route of administration. Patients can administer Adalimumab subcutaneously at home, whereas they must make appointments for IV Infliximab. Another case report by Heffernan et al^4^ reports complete healing of a pyoderma gangrenosum ulcer within 5.5 months of treatment with Adalimumab.

## 5 PATIENT OUTCOME

Our patient was discharged with plans for gentle wound cleansing three times a day. The current plan is for her to continue Adalimumab with a slow prednisone taper and continued monitoring by both dermatology and rheumatology. Now, two months after discharge, the ulcerations are improving. The lesions are smaller in diameter and have more granular tissue. She continues to receive Adalimumab and is on a prednisone taper.

This case highlights the importance of considering pyoderma gangrenosum in a patient with presumed cellulitis who is not responding to antibiotics.

## CONFLICT OF INTEREST

None declared.

## AUTHOR CONTRIBUTION

KMP: wrote manuscript, obtained patient consent, and reviewed manuscript edits. GR: wrote manuscript and reviewed manuscript edits. NR: wrote manuscript, edited manuscript, and acted as corresponding author.

## References

[1] Callen JP , Jackson JM . Pyoderma gangrenosum. Rheum Dis Clin North Am. 2007;33(4):787‐802. 18037117 10.1016/j.rdc.2007.07.016

[2] Binus AM , Qureshi AA , Li VW , Winterfield LS . Pyoderma gangrenosum: a retrospective review of patient characteristics, comorbidities and therapy in 103 patients. Br J Dermatol. 2011;165(6):1244‐1250. 21824126 10.1111/j.1365-2133.2011.10565.x

[3] Regueiro M , Valentine J , Plevy S , Fleisher MR , Lichtenstein GR . Infliximab for treatment of pyoderma gangrenosum associated with inflammatory bowel disease. Am J Gastroenterol. 2003;98(8):1821‐1826. 12907338 10.1111/j.1572-0241.2003.07581.x

[4] Heffernan MP , Anadkat MJ , Smith DI . Adalimumab treatment for pyoderma gangrenosum. Arch Dermatol. 2007;143(3):306‐308. 17372094 10.1001/archderm.143.3.306

